# Comparative Analysis of Robotic Assistive Devices on Paretic Knee Motion in Post-Stroke Patients: An IMU-Based Pilot Study

**DOI:** 10.3390/jfmk11010005

**Published:** 2025-12-24

**Authors:** Toshiaki Tanaka, Shunichi Sugihara, Takahiro Miura

**Affiliations:** 1 Research Center for Advanced Science and Technology (RCAST), The University of Tokyo, Tokyo 113-8656, Japan; 2 Assist General Incorporated Association, 1-55, Kiyota 1-jo 4-chome, Kiyota-ku, Sapporo 004-0841, Japan; 3 Department of Rehabilitation, Sapporo Shuyukai Hospital, 2-1, 2-banchi, 5-jo 6-chome, Shinhassamu, Teine-ku, Sapporo 006-0805, Japan; s-sugi@mtf.biglobe.ne.jp; 4 National Institute of Advanced Industrial Science and Technology (AIST), Kashiwa 277-0882, Japan; miura-t@aist.go.jp

**Keywords:** robotic rehabilitation, exoskeleton, stroke, gait analysis, IMU, RMSE, synchronization, Bland–Altman analysis

## Abstract

**Background**: Robotic assistive devices are increasingly used in post-stroke gait rehabilitation, yet quantitative evaluations of synchronization between human and robotic joint motion remain limited. This study examined gait kinematics in post-stroke hemiplegic patients using two exoskeleton-type devices—HAL^®^ (Cyberdine Inc., Tsukuba, Japan) and curara^®^ (AssistMotion Inc., Ueda, Japan)—based on synchronized IMU measurements. **Methods**: Two post-stroke patients performed treadmill walking under non-assisted and assisted conditions with HAL^®^ and curara^®^. Only the paretic knee joint was analyzed to focus on the primary control joint during gait. Inertial measurement units (IMUs) simultaneously recorded human and robotic joint angles. Synchronization was assessed using Bland–Altman (BA) analysis, root mean square error (RMSE), and mean synchronization jerk (MSJ). The study was designed as an exploratory methodological case study to verify the feasibility of synchronized IMU-based human–robot joint measurement. **Results**: Both assistive devices improved paretic knee motion during gait. RMSE decreased from 7.8° to 4.6° in patient A and from 8.1° to 5.0° in patient B. MSJ was lower during curara-assisted gait than HAL-assisted gait, indicating smoother temporal coordination. BA plots revealed reduced bias and narrower limits of agreement in assisted conditions, particularly for curara^®^. Differences between HAL^®^ and curara^®^ reflected their distinct control strategies—voluntary EMG-based assist vs. cooperative gait-synchronization—rather than superiority of one device. **Conclusions**: Both devices enhanced synchronization and smoothness of paretic knee motion. curara^®^ demonstrated particularly smooth torque control and consistent alignment with human movement. IMU-based analysis proved effective for quantifying human–robot synchronization and offers a practical framework for optimizing robotic gait rehabilitation. The novelty of this study lies in the direct IMU-based comparison of human and robotic knee joint motion under two contrasting assistive control strategies.

## 1. Introduction

In Japan, robotic technology has advanced beyond industrial use into healthcare, including rehabilitation. Since April 2016, robotic rehabilitation has been partially covered by the national health insurance system [1], and the 2020 medical fee revision introduced reimbursement for devices facilitating motor rehabilitation [2]. This has accelerated the clinical application of robotic devices in rehabilitation settings.

Robotic rehabilitation (hereafter, robotic rehab) has attracted attention as a potential solution for stroke-related gait disability. Exoskeleton-type robots, which directly attach to the body and provide power-assisted movements, have been applied for nursing care, rehabilitation, and daily living support. The role of robotic rehab for post-stroke gait disturbance primarily involves assisting hip and knee joint motion of the paretic lower limb [3,4,5,6,7,8,9,10]. Evidence regarding feasibility, efficacy, and safety of the Hybrid Assistive Limb (HAL^®^) has been reported in Japanese and international studies [3,4,8,9]. The two essential factors in HAL^®^-based rehabilitation are training intention (i.e., selecting movement type based on therapist and patient input) and adjustment of control parameters. While therapist expertise is critical, proper control parameter settings are equally important for effective use. However, few studies have conducted detailed analyses of human–robot synchronization, and the effects of misfitting between device and wearer remain unclear.

In Europe and North America, evidence on exoskeleton-based rehabilitation is growing. Randomized controlled trials have demonstrated significant improvements in walking speed and balance compared with conventional therapy [10,11,12]. For example, Buesing et al. suggested that exoskeleton-assisted training may enhance walking ability and long-term neuroplasticity [11]. Such findings highlight the role of robotic rehab not only as an assistive device but also as a neurorehabilitation tool.

However, most previous studies have evaluated either the user’s joint kinematics alone or the robotic device’s joint output alone, and very few have directly examined the real-time synchronization between human and robotic joints [3,12]. Although prior research, including Louie & Eng [12], Kawamoto et al. [3], and Nilsson et al. [6] has demonstrated the clinical effectiveness or control characteristics of robotic gait assistance, these studies did not quantify real-time human–robot synchronization. Similarly, systematic reviews by Mehrholz et al. [10] and soft-exosuit research by Awad et al. [13] have reported improvements in gait performance but did not evaluate the temporal error or interaction metrics (e.g., RMSE, MSJ) between human and robot.

The novelty of the present study lies in simultaneously measuring human knee joint motion and robotic knee joint output using synchronized IMUs during gait, thereby enabling direct comparison of two contrasting assistive control strategies—voluntary EMG-based assist control in HAL^®^ [3,4,5,6,9] and cooperative gait-synchronization control in curara^®^ (AssistMotion Inc., Ueda, Japan) [14,15,16]. To our knowledge, no existing studies have directly assessed human–robot knee joint synchronization in post-stroke hemiplegic patients using time-aligned IMU measurements, making this methodological approach unique.

In this context, the present study aimed to comparatively evaluate two different robotic assistive devices, HAL^®^ and curara^®^, to clarify their respective effects on gait kinematics in two post-stroke hemiplegic patients. Using IMU sensors, real-time synchronization between human and robotic joints was analyzed, focusing on discrepancies in joint axis alignment and their potential implications for fitting optimization.

## 2. Materials and Methods

### 2.1. Participants

Two post-stroke hemiplegic patients participated in this study. Patient A was a male in his 70s who had suffered a left cerebral infarction 30 days after onset. His affected side was the right lower limb, and his Brunnstrom stage for the lower limb was V. The Functional Ambulation Category (FAC) score was 4, and the Functional Independence Measure (FIM) was 125. Patient B was a female in her 40s who had experienced a right cerebral infarction 39 days after onset. Her affected side was the left lower limb, and the Brunnstrom stage for the lower limb was V. The FAC score was 5, and the FIM score was not applicable. Both participants were able to perform independent gait training and met the inclusion criteria for this study. All participants provided written informed consent prior to participation. The study protocol was approved by the Ethics Committee of the University of Tokyo (approval no. 20-210).

This study was designed as an exploratory methodological case study aimed at validating the feasibility of synchronous measurement of human and robotic knee joint motion using IMUs. Because the purpose was methodological verification rather than statistical generalization, a detailed comparison of two carefully documented cases was considered appropriate [12,17]. Future studies with larger cohorts will be necessary to generalize the findings.

### 2.2. Robotic Devices

Two exoskeleton-type gait assist robots were tested: Hybrid Assistive Limb (HAL^®^) and robotic wear curara^®^. For motion analysis, inertial measurement unit (IMU) sensors were attached to both the human body and devices. Additionally, curara^®^ was equipped with an alert system that provided audio and vibratory feedback when preset thresholds of joint angle deviation or heart rate were exceeded.

HAL^®^ and curara^®^ were intentionally selected because they represent two distinct and widely used assistive control strategies in clinical robotic gait rehabilitation: voluntary EMG-based assist control (HAL^®^) and cooperative gait-synchronization control (curara^®^). Comparing these contrasting mechanisms provides important insight into device-specific human–robot interaction characteristics [12,18].

### 2.3. IMU Placement (Figure 1 and Figure 2)

On the human body, sensors were attached 15 cm above and below the lateral knee joint line. On the devices, sensors were attached at the mid-thigh and mid-shank. This allowed synchronized measurement of human and robotic joint kinematics. The lateral thigh and shank positions were selected because they align closely with the anatomical flexion–extension axis of the knee joint, minimizing rotational artifacts and improving sagittal-plane angle estimation accuracy. This placement is consistent with standard IMU gait-analysis protocols [19,20,21].

**Figure 1:**
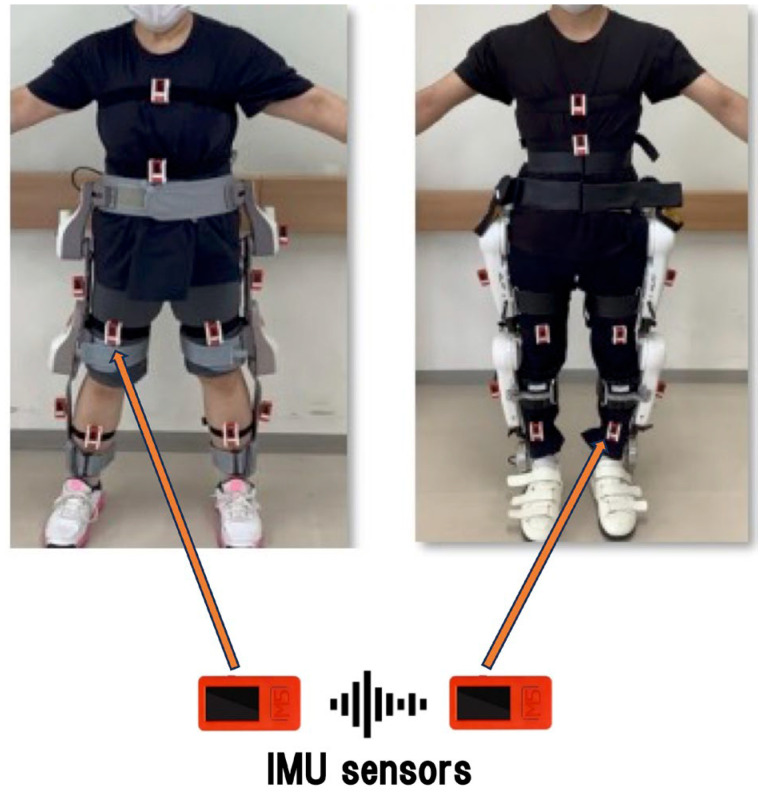
Placement of IMU sensors on human and robotic limbs ((**left**): curara^®^, and (**right**): HAL^®^). Human knee joint angle is measured by sensors placed 15 cm above and 15 cm below the knee joint line; Robotic placement: mid-thigh and mid-shank.

**Figure 2:**
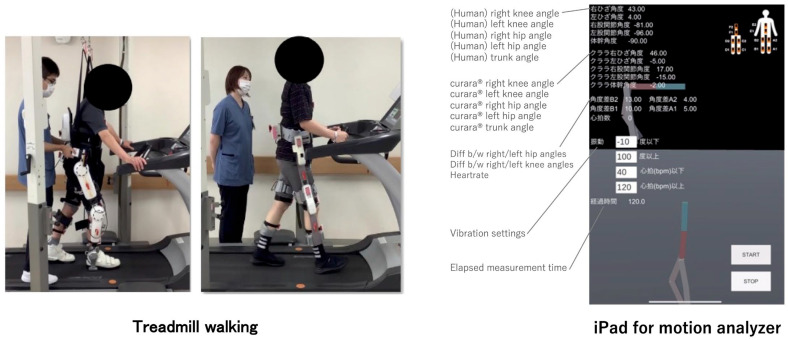
(**Left**) Experimental setup of treadmill walking with exoskeleton-type devices HAL^®^ and curara^®^ under therapist-controlled assistance. (**Right**) Screenshot of the measurement application for each joint angle, reflecting the walking shown in the left figure. This application displays the left/right knee and hip joint angles of the human and the exoskeleton-type device, the angle difference between them, the elapsed measurement time, the visualized IMU sensor positions (A1, A2, …), which correspond to the positions shown in Figure 1, and the simplified motion skeleton. Notations of IMU sensor position were as follows: A1–2 and B1–2: left/right front knee and hip; C1–2 and D1–2: right/left back knee and hip; E1–2 and F1–2: front/back sternum and clavicle. Red and blue-green lines represent positive and negative angle values, respectively.

### 2.4. Procedure (Figure 3)

Patient A and B performed treadmill walking under the following conditions: (1) without robotic assistance, (2) with HAL^®^, and (3) with curara^®^. Each condition lasted 3–5 min at comfortable speed, conducted on separate days. Therapists adjusted assistive parameters based on gait observation. For HAL^®^, control was set at comfortable speed, paretic side CVC mode, non-paretic side CIC mode, with torque ~10 Nm [4]. For curara^®^, unloading was set to none, speed at comfortable pace, and target trajectories at ~50% of normal hip and knee joint motion.

To enable direct comparison between robotic assistive devices, gait data obtained from HAL^®^ and curara^®^ trials were analyzed as separate datasets under identical environmental and treadmill conditions. Both robotic sessions were therapist-controlled, ensuring equivalent assistance intensity and walking speed.

The primary analytical focus was to identify device-specific differences in human–robot synchronization and adaptability during assisted gait. IMUs on the human and robotic limbs were synchronized using a unified 100 Hz timestamp protocol, ensuring accurate temporal alignment during gait measurement [19,20,21].

**Figure 3:**
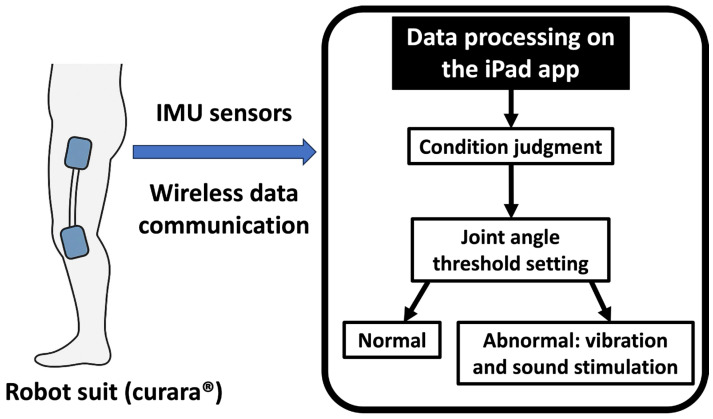
Alert system integrated with curara^®^, providing auditory and vibratory feedback when deviations in joint angle or heart rate thresholds are exceeded. The right panel shows the alarming process: the iPad App (shown in the right panel of Figure 2) processes IMU sensor data, judges the condition, checks the joint angle threshold settings, and then either issues no alert if normal or alerts with vibration and sound stimulation if abnormal.

### 2.5. Data Analysis (Figure 3)

Regarding the installation of IMU sensors on the joint axes of the human and robot suit, measurements were evaluated on the knee joint because it was difficult to install them on the hip joint axis. Only the paretic knee joint was evaluated to focus on the primary control joint relevant to robotic gait assistance. IMU data were recorded and processed to calculate average joint angles and discrepancies. Each trial included eight normalized gait cycles. Stance and swing phases were determined using sagittal-plane angular velocity thresholds and zero-crossing detection based on the shank IMU, following established gait-event identification methods [19,21]. All IMU orientation data were calibrated before analysis, and a fourth-order Butterworth low-pass filter (cutoff frequency 6 Hz) was applied to reduce noise while preserving gait-related movement [19,22,23].

Differences between human and robotic knee angles were analyzed across trials. Because the sample size was extremely small (*n* = 2), statistical hypothesis testing, such as paired *t*-tests was not performed. Instead, descriptive waveform analysis and numerical comparison of RMSE and MSJ values were used. RMSE values are presented both as absolute angular errors (degrees) to facilitate clinical interpretation and as normalized values to enable waveform-level comparison across conditions.

To evaluate the agreement between human gait kinematics and robotic assistance, Bland–Altman (BA) [24,25,26] analysis was performed for both knee joints. In addition to BA analysis, RMSE of the paretic knee joint angle and mean synchronization jerk (MSJ) were calculated to evaluate temporal smoothness and synchronization.

## 3. Results

**Figure 4:**
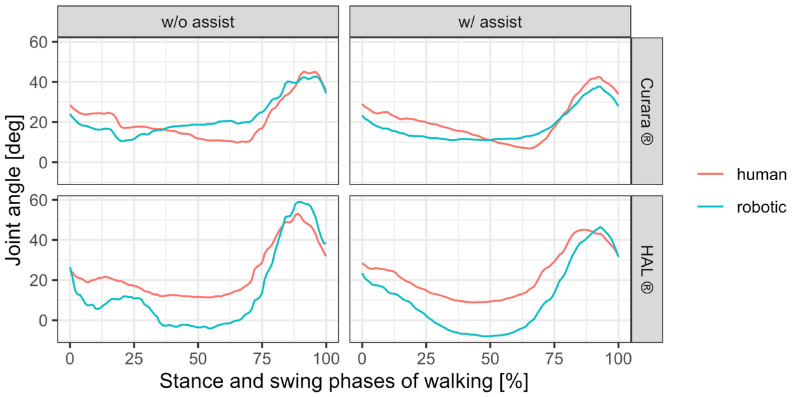
Changes in knee joint angle during gait in patient A, comparing the human and the robot suit.

**Figure 5:**
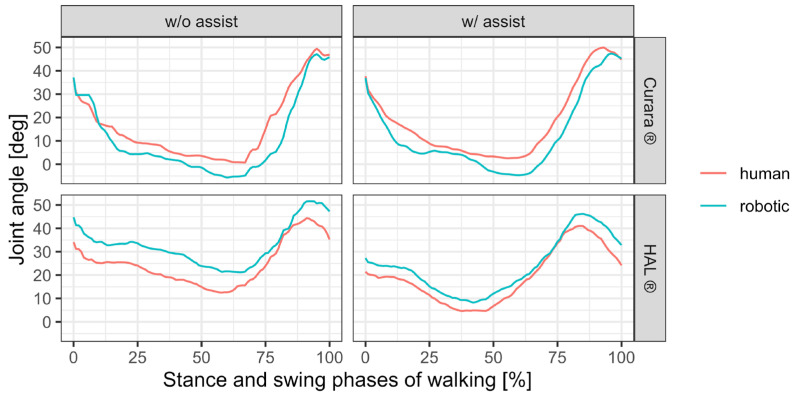
Changes in knee joint angle during gait in patient B, comparing the human and the robot suit.

From the temporal changes in knee joint angles during the stance (approximately 60% of the first half of one gait cycle) and swing phases (approximately 40% of the first half of one gait cycle) of walking, subject A exhibited a larger deviation between the biological and robotic knee joint axes during the transition from stance to swing phase (Figure 4). This difference was particularly pronounced in HAL^®^. For patient B, the deviation in HAL^®^ was slightly less than in curara^®^ during the transition phase, whereas the deviation in HAL^®^ tended to be greater than in curara^®^ during the swing phase when the knee joint reached its maximum angle in Figure 5.

**Figure 6:**
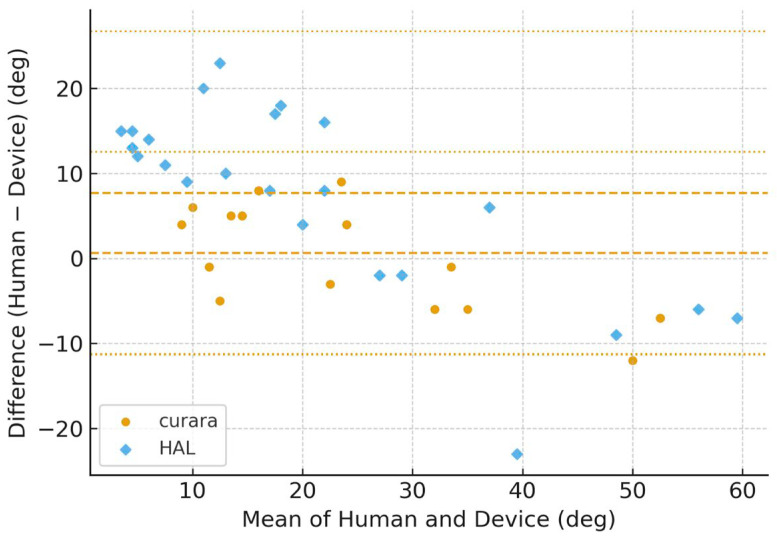
Bland–Altman plots for the paretic knee joint angle during no assistive gait using curara^®^ and HAL^®^ in patient A. Human–robot agreement is shown for curara^®^ (●) and HAL^®^ (◆) in the same panel. Dashed line: mean bias; dotted lines: 95% limits of agreement (LOA). Due to the limited variability of paretic knee joint angles under the No-Assist condition, multiple identical human–robot angle pairs overlap at the same coordinates in the Bland–Altman plots (overplotting), resulting in fewer visible data points despite an equivalent number of underlying samples.

**Figure 7:**
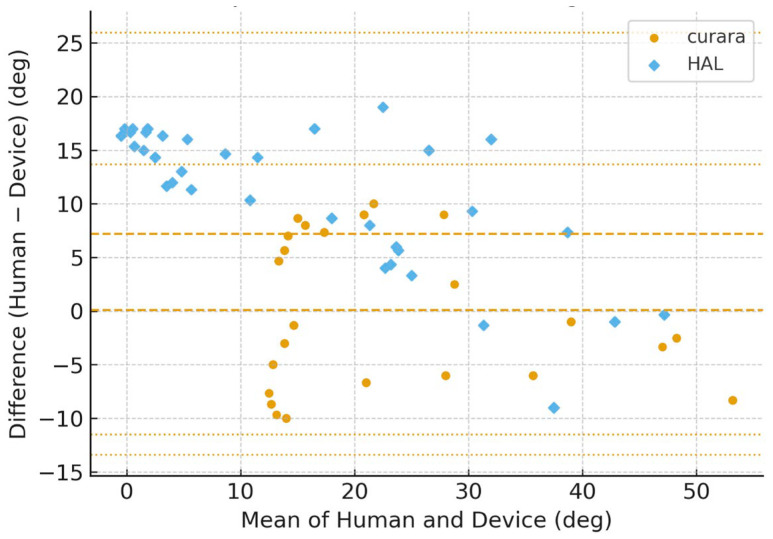
Bland–Altman plots for the paretic knee joint angle during assistive gait (average of Assist 1–3) using curara^®^ and HAL^®^ in patient A. Human–robot agreement is shown for curara^®^ (●) and HAL^®^ (◆). Dashed line: mean bias; dotted lines: 95% limits of agreement (LOA). Due to the limited variability of paretic knee joint angles under the No-Assist condition, multiple identical human–robot angle pairs overlap at the same coordinates in the Bland–Altman plots (overplotting), resulting in fewer visible data points despite an equivalent number of underlying samples.

**Figure 8:**
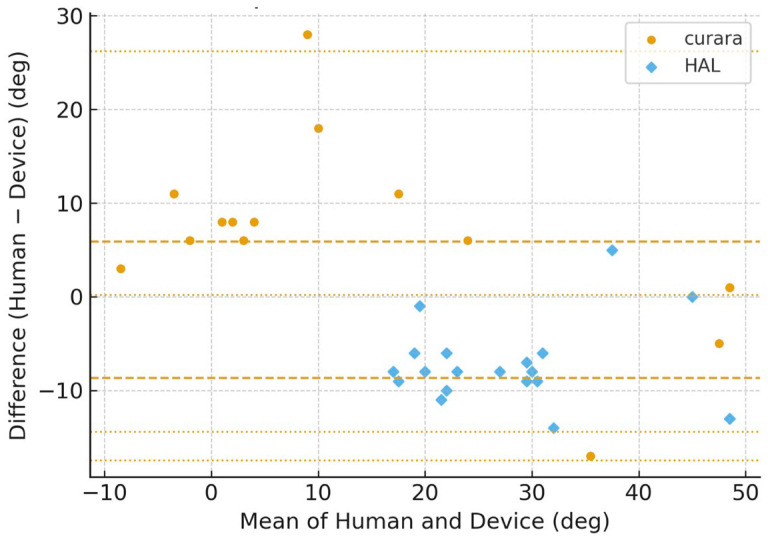
Bland–Altman plots for the paretic knee joint angle during no assistive gait using curara^®^ and HAL^®^ in patient B. Human–robot agreement is shown for curara^®^ (●) and HAL^®^ (◆). Dashed line: mean bias; dotted lines: 95% limits of agreement (LOA). Due to the limited variability of paretic knee joint angles under the No-Assist condition, multiple identical human–robot angle pairs overlap at the same coordinates in the Bland–Altman plots (overplotting), resulting in fewer visible data points despite an equivalent number of underlying samples.

**Figure 9:**
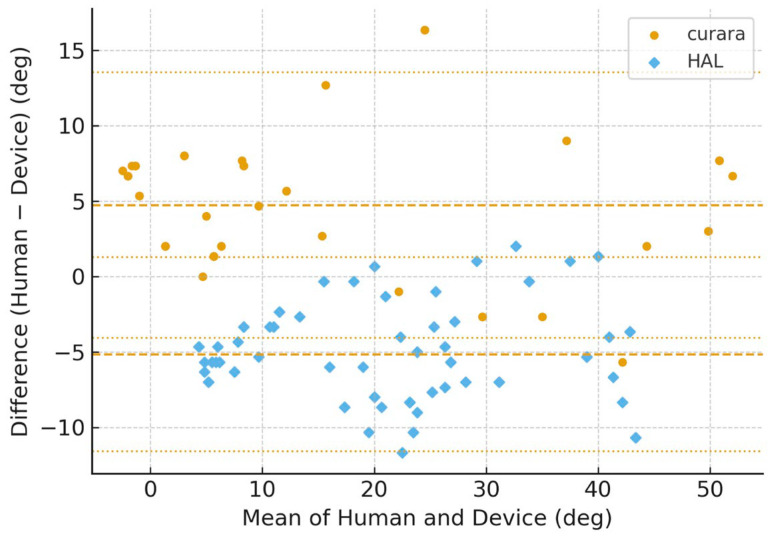
Bland–Altman plots for the paretic knee joint angle during assistive gait (average of Assist 1–3) using curara^®^ and HAL^®^ in patient B. Human–robot agreement is shown for curara^®^ (●) and HAL^®^ (◆). Dashed line: mean bias; dotted lines: 95% limits of agreement (LOA). Due to the limited variability of paretic knee joint angles under the No-Assist condition, multiple identical human–robot angle pairs overlap at the same coordinates in the Bland–Altman plots (overplotting), resulting in fewer visible data points despite an equivalent number of underlying samples.

Figure 6, Figure 7, Figure 8 and Figure 9 show the Bland–Altman plots of the knee joint angle trajectories of the patient for a comparison between the human limb and HAL^®^ device during the stance and swing phases. Figure 6 and Figure 7 showed patient A, and Figure 8 and Figure 9 showed patient B, allowing direct comparison between both subjects. To avoid misunderstanding, it should be noted that the No-Assist condition did not contain fewer data samples. On the paretic side, knee joint angles exhibited limited variability, producing many identical human–robot angle pairs. In the Bland–Altman analysis, identical pairs are plotted at the same coordinates; thus, multiple observations overlap (overplotting), making the visible number of points appear smaller despite an equivalent amount of underlying data. This behavior is a known characteristic of Bland–Altman plots and is reported in previous methodological studies [25,26,27]. The LOA indicates the range within which 95% of the differences are expected to fall, representing the degree of agreement between the robotic and human measurements.

The BA plots demonstrated distinct patterns between curara^®^ and HAL^®^. For the knee joint, curara^®^ showed a small positive bias (+2.2°) in the assisted condition with narrow LOA (−10.8° to +15.2°), indicating high agreement with human motion. In contrast, HAL^®^ exhibited a negative bias (−5.3°) with wider LOA (−38.5° to +27.9°), suggesting lower agreement. Overall, curara^®^ tended to produce more consistent results with human gait compared to HAL^®^, particularly for the knee joint.

The reduction in both RMSE and MSJ values under assisted conditions provides quantitative confirmation of improved synchronization and smoother joint motion, supporting the qualitative findings observed in the waveform analysis. Comparative analysis of RMSE and MSJ in patients A and B demonstrated that both showed improved waveform smoothness under robotic assistance. Table 1 summarizes the quantitative results of RMSE and MSJ for the paretic knee joint in both participants (patient A and B) during No-Assist and Assist walking using HAL^®^ and curara^®^.

For participant patient A, RMSE decreased from 0.148° (No-Assist) to 0.103° (Assist) with HAL^®^, and from 0.136° to 0.087° with curara^®^. Similarly, for participant patient B, RMSE decreased from 0.162° (No-Assist) to 0.112° (Assist) with HAL^®^, and from 0.142° to 0.098° with curara^®^. MSJ, representing motion smoothness, also showed consistent reductions: patient A exhibited a decrease from 0.018 to 0.011 with HAL^®^, and from 0.017 to 0.009 with curara^®^, while patient B showed reductions from 0.020 to 0.013 with HAL^®^, and from 0.018 to 0.010 with curara^®^. These results indicate that robotic assistance improved both joint motion agreement and movement smoothness across conditions. Moreover, both assistive devices improved paretic knee motion during gait. RMSE decreased from 7.8° to 4.6° in patient A and from 8.1° to 5.0° in patient B. Moreover, both assistive devices improved paretic knee motion during gait. RMSE values of patient A improved from 7.8° to 4.6°, and patient B improved from 8.1° to 5.0° between No-Assist and Assist conditions. Abnormal gait was observed in the patient without robotic assistance, including insufficient knee extension during stance and reduced hip extension at stance-to-swing transition. With robotic assistance, partial normalization occurred; however, variability remained. Among three assisted trials, at least one showed near-normalization of joint motion.

According to the Alert system, the curara^®^ alert system could detect abnormal IMU deviations and execute device stopping within approximately 30 ms, demonstrating the feasibility of real-time monitoring rather than evaluating clinical safety outcomes.

## 4. Discussion

### 4.1. Quantitative Metrics of Synchronization (RMSE and MSJ)

These BA analyses provide further insight into the agreement between human gait and robotic assistance. Importantly, these observed differences should not be interpreted as evidence of device superiority but rather as reflections of distinct assistive control strategies and human–robot interaction characteristics. In the Bland–Altman analysis, narrower LOA values indicate better agreement and thus higher clinical applicability, whereas wider LOA reflects possible misalignment or device-specific limitations [25,28,29]. Moreover, the LOA values observed in this study can be interpreted relative to clinically acceptable error ranges, providing a practical index for evaluating usability and patient safety during gait rehabilitation. Narrow LOA indicates accuracy that is acceptable in clinical practice, whereas wider LOA suggests the need for further control adjustments or individualized calibration. The results suggest that curara^®^ achieves closer alignment with human knee motion, reflecting its potential effectiveness for knee joint rehabilitation. The limits of agreement (LOA) represent the range within which 95% of the differences between human and robotic knee joint angles are expected to fall, thereby indicating the degree of agreement and potential clinical acceptability of the robotic assistance.

The consistent reduction in RMSE and MSJ values across conditions indicates enhanced temporal and kinematic synchronization. These results demonstrate that quantitative IMU-based metrics can sensitively detect improvements in gait smoothness and coordination under robotic assistance. This improvement was more pronounced in curara^®^ for patient A and in HAL^®^ for patient B, reflecting possible differences in residual motor control and device adaptation. The consistent reduction in MSJ indicates smoother motion during robotic-assisted walking, which aligns with previous studies reporting that exoskeleton assistance reduces gait variability and improves rhythmicity [11,13,14,15,22,30].

The distinct device-dependent patterns may stem from the underlying control principles: curara^®^ employs trajectory-based torque control designed to reproduce physiological motion curves, whereas HAL^®^ utilizes bioelectric feedback, adapting more directly to user-generated voluntary muscle activity. Similar findings were reported by Tanaka et al. (2022), who observed that modulating assistive load levels in an exoskeleton-type robotic device significantly affected gait symmetry and adaptation speed during stroke rehabilitation [31].

The present study integrated qualitative waveform interpretation with quantitative indicators (RMSE and MSJ). This dual approach is consistent with previous robotic gait-analysis frameworks and strengthens the interpretability of synchronization findings [18,19,20,22]. Differences observed between HAL^®^ and curara^®^ should not be interpreted as device superiority but as reflections of differences in their control strategies—voluntary EMG-based assist control for HAL^®^ and cooperative gait-synchronization control for curara^®^ [4,5,14,15,16,18].

### 4.2. Device Alignment and Clinical Implications

Previous studies have also reported that misalignment of exoskeleton joint axes relative to anatomical joints can increase muscle loading, interaction torque, and user discomfort [28,29,32]. These findings support the present results regarding joint mismatch and its functional implications. In design studies as well, mitigating misalignment is considered crucial for comfort and usability [33,34]. However, this study is based on a single case, and thus the generalizability of the findings is limited. To overcome this limitation, future research should expand the sample size and incorporate multicenter trials as well as comparisons with motion capture systems.

Randomized controlled trials of exoskeletal-assisted walking have been conducted in Western populations, including a JAMA clinical trial in veterans with paralysis [35]. Moreover, meta-analyses in stroke cohorts suggest that robotic exoskeleton training improves walking-related outcomes compared with conventional therapy [36,37]. This study provides an important initial report that positions Japan’s assistive robotic technologies within the context of international evidence.

According to the clinical implications, misalignment between robotic and anatomical joint axes is a major cause of discrepancies, which may reduce gait efficiency, cause discomfort, or increase fall risk [28,29,30,31,32,33]. Proper fitting adjustments and real-time dynamic evaluation are essential. Individualized tuning of control parameters based on patient muscle strength, gait ability, and characteristics may optimize rehabilitation outcomes. IMU-based monitoring offers a practical, low-burden approach for adjustment and progress tracking [19,20,21].

The variability observed in knee-angle synchronization in this study may also be partially attributable to residual misalignment between anatomical and mechanical joint centers. Prior biomechanical research has shown that even small alignment deviations can influence joint-angle estimation and torque output; therefore, alignment sensitivity should be considered in interpreting synchronization results [20,28]. In addition, the use of IMU-based methods is supported by previous sensor-fusion and calibration studies demonstrating that low-cost wearable IMUs can reliably capture joint motion when appropriate filtering and calibration techniques are employed [19,20,21]. Furthermore, comprehensive methodological reviews have highlighted the clinical applicability of IMU-based joint kinematic assessments, particularly in rehabilitation contexts where traditional motion capture systems may be impractical or unavailable [19,38].

### 4.3. Limitations and Future Work

For the Limitations, the study was limited to two stroke patients. Larger-scale studies are needed. Future work should compare IMU data with motion capture for accuracy validation and develop mechanisms to correct joint axis misalignment. Although both participants demonstrated similar trends, subtle asymmetries between right and left hemiplegia may reflect individual neural compensation mechanisms that warrant further study.

Because the sample size was extremely small (*n* = 2), these findings should be interpreted as case-specific observations rather than generalizable outcomes. Individual differences such as age, severity of paresis, and compensatory gait patterns may have influenced synchronization trends [30]. Nevertheless, detailed methodological case studies are essential when validating new measurement frameworks in rehabilitation robotics. Since synchronous human–robot joint measurement has rarely been investigated, individual-level evaluation was required to verify feasibility and data quality before proceeding to large sample trials [17,19].

## 5. Conclusions

In conclusion, this two-case study demonstrated that robotic assistance using HAL^®^ and curara^®^ improved knee joint synchronization and motion smoothness in post-stroke gait rehabilitation. Quantitative IMU-based indices (RMSE and MSJ) effectively captured these improvements and confirmed robotic assistance’s superior adaptability. These findings validate the clinical utility of IMU-based assessment for optimizing robotic rehabilitation parameters and support its broader application in individualized therapy design.

Importantly, the novelty of this study lies in the direct comparison of human and robotic knee joint angles using synchronized IMU sensors, enabling a quantitative evaluation of human–robot interaction under two distinct control strategies—voluntary EMG-based assist control (HAL^®^) and cooperative gait-synchronization assistance (curara^®^) [4,16,38].

Although the sample size was extremely small (*n* = 2), this methodological case study provides foundational evidence supporting the feasibility of synchronous IMU-based gait assessment, which is necessary before progressing to larger-scale trials [17,19].

Future research should expand participant numbers, refine fitting mechanisms, and validate findings using advanced motion capture systems. Additionally, future work should incorporate alignment-compensation algorithms and multi-joint synchronization analysis to further address concerns arising from residual misalignment between anatomical and robotic joint axes [20,32,33,39].

## Figures and Tables

**Table 1:** Mean RMSE and MSJ of paretic knee joint motion during one gait cycle under robotic-assisted gait conditions.

Participant	Device	Condition	RMSE [°]	MSJ [°^2^/s^6^]
Patient A	HAL^®^	No-assist	0.148	0.018
		Assist	0.103	0.011
	curara^®^	No-assist	0.136	0.017
		Assist	0.087	0.009
Patient B	HAL^®^	No-assist	0.162	0.02
		Assist	0.112	0.013
	curara^®^	No-assist	0.142	0.018
		Assist	0.098	0.01

RMSE: Root Mean Square Error; MSJ: Mean Squared Jerk. Lower values indicate higher agreement and smoother knee motion.

## Data Availability

The datasets analyzed during this study are available from the corresponding author upon reasonable request.
